# Long-term follow-up after surgery in localized laryngeal amyloidosis

**DOI:** 10.1007/s00405-016-4061-y

**Published:** 2016-05-07

**Authors:** Aldert J. C. Hazenberg, Bouke P. C. Hazenberg, Frederik G. Dikkers

**Affiliations:** 1 Department of Otorhinolaryngology, Refaja Hospital Stadskanaal, Boerhaavestraat 1, 9501 HE Stadskanaal, The Netherlands; 2 Departments of Rheumatology and Clinical Immunology, University of Groningen, University Medical Center Groningen, Groningen, The Netherlands; 3 Department of Otorhinolaryngology, University of Groningen, University Medical Center Groningen, Groningen, The Netherlands

**Keywords:** Long-term follow-up, Surgery, Localized laryngeal amyloidosis

## Abstract

To study effectiveness of surgery and watchful waiting in localized laryngeal amyloidosis, retrospective case series. This retrospective study comprises all consecutive patients with localized laryngeal amyloidosis surgically treated in a tertiary hospital between 1994 and February 2016. Recurrence rate, revision surgery, progression to systemic amyloidosis, and changes in voice were monitored yearly. Eighteen patients were included. Seven women and eleven men had a median age 50 years (range 21–77 years) and median follow-up 6.4 years (2.4–17 years). Amyloid was located in subglottis (5), glottis (8), false vocal folds (8) and other supraglottic areas (5), in more than one laryngeal region (13) and bilaterally (12). Cold steel excision was used at the glottis; CO_2_ laser excision, sometimes assisted by microdebrider, at other laryngeal areas. Eleven patients needed revision surgery, ten within the first 4 years after surgical treatment. One patient needed his first revision surgery after 11 years. Five patients needed a second revision within 6 years after initial diagnosis. Two patients needed a third revision. Indications for first revision surgery were progression (8) with dysphonia (7), dyspnea (2), dysphagia (1), exclusion of malignancy (1), and aphonia (1). No patient developed systemic amyloidosis during follow-up. Although local progression of amyloid necessitates revision surgery once or twice in the first 4–6 years, progression slows down thereafter. Late progression, however, remains possible.

## Introduction

Localized laryngeal amyloidosis is a benign tumorous disease of the laryngeal region. It consists of amorphous extracellular deposits of AL amyloid fibrils. The AL amyloid is derived from immunoglobulin light chains produced by clonal plasma cells nearby [1]. Localized amyloidosis differs from systemic amyloidosis. In systemic amyloidosis, the soluble precursor protein is produced elsewhere in the body (e.g., liver or bone marrow), released into the circulation and deposited as insoluble amyloid fibrils throughout the body [2]. Although rare, laryngeal amyloidosis can be a disease manifestation of systemic AL amyloidosis [3, 4] or of systemic AApoAI amyloidosis [5]. (The type of amyloid is designated as a capital A followed by the characteristic precursor protein [2]; e.g., AL amyloidosis is derived from *κ* or *λ* immunoglobulin light chains and hereditary type AApoAI amyloidosis from mutated apolipoprotein AI [2]). Therefore, in the clinical approach of laryngeal amyloidosis, investigations are directed to make systemic amyloidosis highly unlikely before concluding localized laryngeal amyloidosis [3].

Aim of effective therapy of localized laryngeal amyloidosis is to obtain a long disease-free interval with preservation of a good voice, of normal swallowing, and absence of dyspnea. Therapeutic options are various modalities of surgery [3, 6–12]. In rare cases, irradiation has been advocated [13, 14]. In 2004 we proposed a therapeutic strategy of suspension microlaryngoscopy guided both by symptoms (e.g., dysphonia or dyspnea) and signs (relevant stenosis) [3]. Complete excision is preferred. Debulking of the amyloid deposits is only performed if radical excision would increase the risk of scarring, dysphonia, or dysphagia. The surgical modalities are adapted to the localization; cold steel excision is used for precise work in critical anatomic sites such as the true vocal cords or in case of small deposits. CO_2_ laser and microdebrider are used in other parts of the larynx or in bulky amyloid. If complete excision proved to be impossible without high morbidity, patients were followed on a yearly basis.

Aim of this study was to investigate the long-term effectiveness of our approach in localized laryngeal amyloidosis in terms of disease progression, recurrence rate after surgery, progression to systemic amyloidosis and postoperative voice characteristics 10 years after our initial study [3].

## Materials and methods

### Study design, setting and patient selection

This longitudinal observational cohort study comprised all consecutive patients with localized laryngeal amyloidosis referred to our tertiary hospital between 1994 and February 2016. All patients were included who presented with localized laryngeal amyloidosis and were treated with surgery. Excluded were patients with systemic amyloidosis with involvement of the larynx or patients not eligible for systematic follow-up every 6–12 months. After a follow-up period of 10 years patients without symptomatic amyloidosis were dismissed.

Due to the observational nature of this longitudinal study, according to Dutch law informed consent and permission of the local ethics committee are not required.

### Variables

The localization of the amyloid in the larynx and modalities of surgery were described. During the follow-up period the local progression and recurrence rate, number of revision surgery procedures, progression to systemic amyloidosis, and changes in voice were monitored yearly, except in the first year when patients were monitored more frequently.

### Local laryngeal evaluation and voice analysis

The larynx was evaluated by videolaryngostroboscopy (digital video stroboscope, model 9100B, KayPentax, Lincoln Park, NJ, USA) with a 90° Von Stuckrad telescope (R. Wolf, Knittlingen, Germany). The voice was analyzed with phonation time, phonetography (voice range profile), GRBAS scoring system and Voice Handicap Index-30 (VHI-30). Phonation time and phonetography are parameters to evaluate the voice [15]. The GRBAS scoring system is a perceptual, subjective evaluation of the voice [16]. The VHI-30 is an assessment of the subjective handicap of the voice [17]. It ranges from 0 (no complaints) to a maximum of 120. A change of more than 14 is significant in the Dutch version of the VHI [17].

### Systemic clinical evaluation

Systemic amyloidosis was made unlikely in each patient by thorough clinical evaluation. A standard protocol was used to evaluate heart, liver, kidney, bone marrow, and peripheral and autonomic nervous system [3, 5]. Systemic amyloidosis was looked for using biopsies of abdominal fat, rectum or bone marrow, along with whole body ^123^I-labeled serum amyloid P component scintigraphy (^123^I-SAP scan), a method used for imaging visceral amyloid deposits [18].

### Surgical treatment

As surgical modalities during suspension microlaryngoscopy, cold steel surgery, CO_2_ laser excision, a microdebrider, or a combination of these techniques were used. All patients underwent surgery under general anesthesia with orotracheal intubation or high frequency jet ventilation. Planned staged interventions on each side of the glottis with an interval of 3–6 months, to prevent webbing, were counted as one single intervention and not as two interventions.

### Statistical analysis

Statistical analysis was performed using Graphpad Prism version 5.04 for Windows, (Graphpad Software, San Diego CA, USA). The Kaplan–Meier curve was used to calculate the percentage of patients who needed revision surgery during follow-up.

## Results

### Patient characteristics

Twenty-two patients with laryngeal amyloidosis were referred to our tertiary center between 1994 and 2015. Eighteen patients could be included. Median age of the 18 included patients (seven women and eleven men) was 50 years at first presentation (range 21–77 years). Five patients have already been reported in our initial report [3] and were now re-evaluated after longer follow-up. Characteristics of all patients are shown in Table 1.

**Table 1:** Localization and surgical data of the patients with localized laryngeal amyloidosis

N	Sex	Age	I. No	Localization					Indic	Surgical modality			Aim
				SG	FVF	TVF	Sub	O		BD	Laser	MD	
1	F	68	IS	–	–	B	–	–	II, III	B	–	–	Ex
			R1	–	–	L	–	–	I, II, III	L	–	–	Ex
2	F	75	IS	–	–	–	B	–	III	B	–	–	Ex
3	M	77	IS	–	–	L	–	–	III, VI	–	L	–	Ex
			R1	R	–	L	–	–	I, III	–	L	–	Ex
			R2	L	L	–	–	–	I, II, III	–	L	–	Ex
4	M	54	IS-a	–	–	B	B	–	III	L	–	–	Db
			R1	–	–	L	L	–	III	–	L	–	Ex
5	M	21	IS	B	B	–	–	^a^	III	–	–	–	NA
			R1	B	B	–	–	–	I, III	–	B	B	Ex
			R2	L	R	–	–	–	I, III	–	B	B	Ex
			R3	–	B	–	–	–	III	–	B	–	Ex
6	M	53	IS	R	–	–	–	–	III	–	–	–	Ex
			R1	R	–	–	–	–	I, III	–	R	R	Ex
7	M	55	IS	–	R	–	–	–	III	–	R	R	Ex
8	M	55	IS	B	–	–	–	–	III	–	B	B	Ex
9	M	45	IS	–	–	B	–	–	III	–	R	–	Db
10	F	42	IS	–	–	–	B	–	III	B	–	–	Db
11	M	49	IS	B	B	B	–	–	III	B	–	–	Db
			R1	–	B	–	–	–	I	–	B	–	Db
			R2	–	B	–	–	–	I	–	B	–	Ex
12	M	72	IS	–	L	–	–	–	III	L	–	–	Db
13	F	51	IS	–	R	–	–	–	III	R	–	–	Ex
			R1		R	–	–	–	III	–	R	–	Ex
14	F	40	IS	–	B	–	–	–	III	B	–	–	Ex
15	F	39	IS-a	–	–	B	B	–	III	B	–	–	Db
			R1	–	–	B	B	–	I, IV	R	–	–	Ex
16	F	41	IS	–	L	B	L	^b^	II, III	B	–	–	Db
			R1	–	L	L	L	–	I, II, III	TVF	FVF	–	Db
			R2	–	–	–	R	–	I, II, III	–	B	–	Ex
			R3	–	–	B	–		I, II, III		B		Db
17	M	23	IS	R	R	–	–	–	III	–	R	–	Ex
			R1	R	R	–	–	–	V	–	R	–	Ex
			R2	R	R	R	–	–	I, II	–	R	–	Ex
18	M	49	IS-a	–	–	B	–	–	III	L	–	–	Db
			IS-b						III	R	–	–	Db
			R1	–	–	B	B	–	I, VI	B	–	–	Db

*N* patient number, *I. no* intervention number, *Age* age at presentation, *Indic.* indication for surgery, *I* progression of disease, *II* dyspnea, *III* dysphonia, *IV* aphonia, *V* dysphagia, *VI* suspected malignancy, *Aim* surgical aim, *SG* supraglottic other than false vocal fold, *FVF* false vocal fold, *TVF* true vocal fold, *sub* subglottis, *O* outside the larynx, *BD* blunt dissection, *Laser* CO_2_ laser, *MD* microdebrider, *M* male, *F* female, *R* right side, *L* left side, *B* bilateral, *IS* initial surgery, *R1* first revision, *R2* second revision, *R3* third revision, *IS-a* planned staged initial surgery, first stage, *IS-b* planned staged initial surgery, second stage, *Ex* excision, *NA* not available, *Db* debulking, Patients number 8, 15–18 have been described earlier as patient number 5, 3, 2,1, and 4, respectively, in Bartels et al. [3]

^a^Amyloid in oropharyx

^b^Amyloid in eyelids and conjunctivae

Four patients were excluded. The first patient was lost during follow-up. The second patient was excluded because she was treated with postoperative irradiation after initial surgery. The third patient presented for the first evaluation 10 years after therapeutic suspension microlaryngoscopy elsewhere, and was not operated during follow-up. The fourth was excluded because she did not present with localized laryngeal amyloidosis, but multifocal amyloidosis in the nasopharynx, the larynx and tracheobronchial tree necessitating tracheotomy.

### Localization of laryngeal amyloid

Patient characteristics are presented in Table 1. The sites where amyloid in the larynx was found are displayed in Tables 1 and 2. Twelve patients had bilateral deposition of amyloid. Amyloid deposition pattern at initial presentation was multifocal in four patients (Nos. 4, 5, 11 and 16) and discretely unifocal in four patients (Nos. 6, 10, 14, and 15). A striking observation was the fact that in some patients (e.g. Nos. 5 and 16) recurrent amyloid bulk shifted from one side to the other, or from one part of the larynx to another.

**Table 2:** Amyloid localization and indications for surgery

	1st surgery (*N* = 18)	1st revision (*N* = 11)	2nd revision (*N* = 5)	3rd revision (*N* = 2)	Residual disease^a^ (*N* = 12)
Localization					
Subglottis	5	4	1	0	4
Glottis	8	6	1	1	5
False vocal folds	8	5	4	1	1
Other supraglottic areas	5	4	3	0	4
Indications					
Progression	NA	8	5	1	
Dyspnea	2	2	3	1	
Dysphonia	15	7	3	2	
Aphonia	0	1	0	0	
Dysphagia	0	1	0	0	
Possible malignancy	1	1	0	0	

Notice that patients can have amyloidosis or recurrence at more than one laryngeal area

*NA* not applicable

^a^Two patients had residual disease in two laryngeal regions

Detailed information of the initial distribution of amyloid could not be retrieved in the remaining patients. These patients were referred to our center after the first diagnostic endoscopy performed elsewhere.

### Surgical treatment

The indications for surgery are listed in Tables 1 and 2. The aim of surgery was either excision or debulking of the amyloid deposits. Cold steel or CO_2_ laser excision was used as surgical modality, in five cases supported by use of a microdebrider. In one case (No. 18), a staged intervention was performed for the left and right side of the glottis separately to prevent webbing. In two other cases with a planned staged intervention (Nos. 4 and 15), the second intervention was cancelled because of a good postoperative voice.

### Investigation of systemic amyloidosis

None of the patients developed systemic amyloidosis during follow-up. In three cases suspicion of systemic amyloidosis persisted despite negative first tests (case 3, 5, and 16). An elevated immunoglobulin free light chain serum level (reference values: *κ* < 20.0 mg/l, *λ* < 32.0 mg/l) was found in two patients (No. 3 and 5).

A 77-year-old man (No. 3) had an elevated serum level of ë free light chain (52.0 mg/l) and a small ë-positive plasma cell clone in the bone marrow. He needed revision surgery twice because of severe dysphonia and progression of the local amyloid bulk in both vocal folds.

A 21-year-old man (No. 5) with amyloid deposits of the left posterior faucial pillar, right oropharyngeal wall, epiglottis and both false vocal folds, had an elevated serum level of *λ* free light chain (72.7 mg/l) and a small *λ*-positive plasma cell clone in his bone marrow. He needed revision surgery three times. The third revision was not for amyloid but because of synechia of the false vocal folds just superior of the anterior commissure.

A 41-year-old woman (No. 16) had not only AL amyloid deposits in the left false vocal fold and the left subglottis, but also in both eyelids and conjunctivae. She needed revision surgery three times because of local progression of the amyloid, leading to dyspnea and dysphonia.

Systemic amyloidosis was not found on repeated thorough investigations, including ^123^I-SAP scintigraphy and analysis of abdominal fat and rectum tissue, in any of the three patients.

### Revision surgery during follow-up

**Fig. 1:**
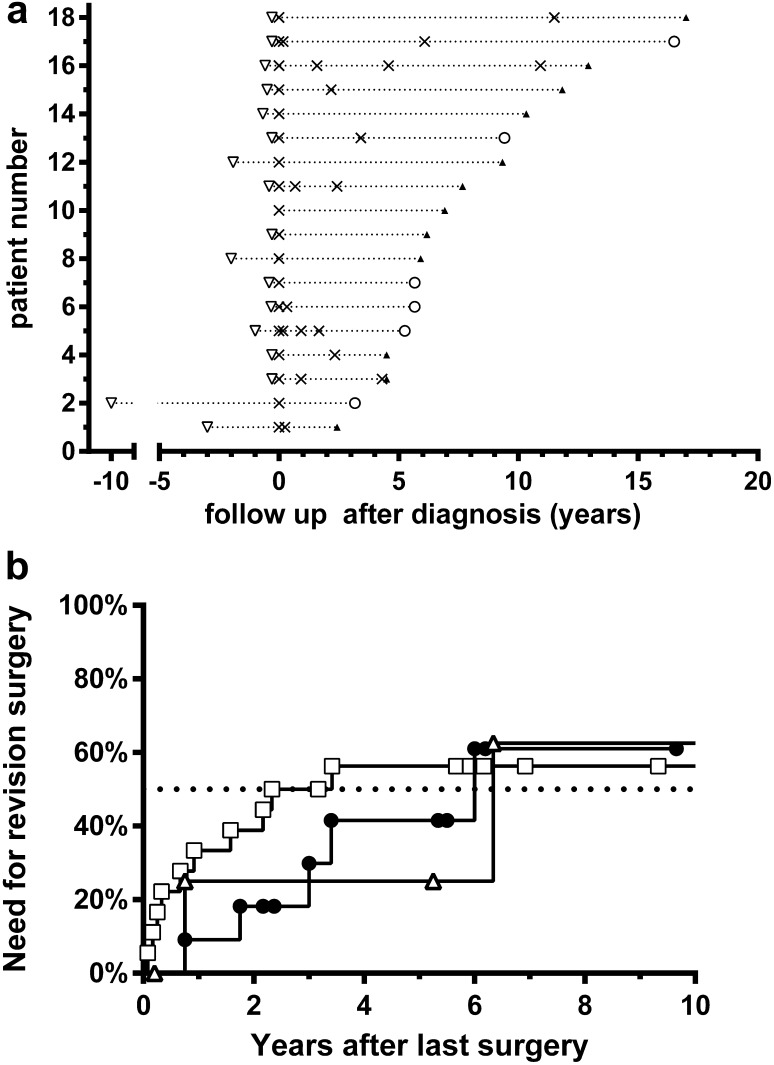
**a** Follow-up of all patients after initial surgical treatment of localized laryngeal AL amyloid. For each individual patient (number corresponds with Table 1) the time interval is displayed of first symptoms (*open triangle*) to initial surgical treatment (*t* = 0) and thereafter each surgical intervention (X) and duration of follow-up (*dotted line*). At the end of each *line* is displayed the presence (*closed triangle*) or absence (*open circle*) of amyloid residual disease as observed at the last visit. **b** Need for revision surgery after last surgical treatment of localized laryngeal amyloid. Kaplan–Meier curves show the need for first revision surgery (*open boxes*, *N* = 18), for second revision surgery (*closed circles*, *N* = 11), and for third revision surgery (*open triangles*, *N* = 5) during 10 years after the last surgical procedure. Subjects censored at the end of their follow-up are located on a *horizontal* part of the *curves*. Subjects who had that revision surgery as event are located on *top* of *vertical* parts of the *curves*. The *vertical axis* shows the actuarial risk of revision surgery as percentage of all patients who had been treated with surgery. The *dotted line* represents the 50 % risk of revision surgery

Follow-up of the individual patients is shown in Fig. 1a. Median follow-up was 78 months (range 29–204 months). Eleven patients needed revision surgery. Five of them needed a second revision within 6 years after initial surgical treatment (Fig. 1b). Two patients, No. 5 and 16, needed a third revision 9 months and 6.3 years after the second revision, respectively. One patient underwent his first revision after 11 years of follow-up because of unexpected and rapidly progressive dysphonia and to rule out malignancy (No. 14). At the last visit 12 of the 18 patients still had clinically indolent residual amyloid without signs of progression (Table 2; Fig. 1a).

### Voice analysis

GRBAS score, phonetographic parameters, aerodynamic values and VHI-30 were available only in a limited number of cases as shown in Table 3. The numbers were too small for statistic analysis. Phonetographic parameters and aerodynamic parameters (phonation times on /a/, /s/ and /z/) were available of 8 patients before initial surgery, 11 prior to revision surgery and of 17 patients at the last visit. A positive trend was present in GRBAS, melodic range, and phonation times. In the few patients of whom VHI-30 values were present, there was a clinically significant improvement.

**Table 3:** Voice characteristics in patients with localized laryngeal AL amyloidosis

	Preoperative (*N* = 18)	Before 1st revision (*N* = 11)	At last visit (*N* = 18)	Normal values
GRBAS score	(*N* = 7)	(*N* = 8)	(*N* = 14)	
G	2 (1–3)	2 (2–3)	1 (1–2)	0
R	2 (1–3)	2 (1–3)	1 (0–1)	0
B	1 (0–3)	0 (0–3)	1 (0–2)	0
A	0 (0–2)	0 (0–1)	0 (0–1)	0
S	0 (0–2)	1 (0–2)	0 (0–1)	0
Phonetographic parameters	(*N* = 8)	(*N* = 11)	(*N* = 17)	
Melodic range (semitones)	18 (10–28)	24 (14–30)	25 (14–32)	30
Dynamic range (dB)	25 (20–30)	20 (16–23)	26 (20–35)	40
Phonation time (s)				
/a/	13 (1–20)	20 (8–23)	16 (12–21)	20
/z/	14 (11–22)	16 (10–19)	17 (12–18)	25
/s/	17 (11–19)	15 (8–22)	14 (12–20)	45
Handicap	(*N* = 4)	(*N* = 5)	(*N* = 11)	
Voice handicap index-30	51 (26–51)	34 (33–51)	22 (1–35)	<10

All values are displayed as median and interquartile range (25–75 % percentile)

*G* overall grade or degree, *R* roughness, *B* breathiness, *A* asthenicity, *S* strained quality

## Discussion

Long-term effectiveness of our surgical approach in localized laryngeal amyloidosis was studied. This study is the largest cohort study of protocol-based [3] monitoring of patients with localized laryngeal amyloidosis having a median follow-up of more than 5 years. Implicitly, watchful waiting was performed in cases with residual disease. Amyloid recurred locally in all patients after initial surgery, but disease progression stopped after 7 years after the last surgery in all but one of the patients. Eleven patients needed revision surgery. Five patients needed a second revision, two needed a third revision. Postoperative voice of most patients, when recorded, was only mildly affected. None of the patients developed systemic amyloidosis during follow-up.

Clinical follow-up by a laryngologist is based on symptoms and signs. The cumulative follow-up of nearly 145 patient-years in this group of 18 patients showed residual amyloid after first surgery in all patients. However, a need for revision surgery was present in only two-thirds of the patients within 4 years after initial surgery (Fig. 1b). A need for a second revision surgery was seen in almost half of the patients within 6 years after initial surgery. Two patients needed a third revision surgery within 7 years, respectively, after second surgery (Fig. 1b).

Presence of residual amyloid after more than 10 years follow-up has been described [10–12, 19–21]. Need for revision surgery after more than 10 years, as in patient No. 14 and 16, is rarely indicated [10, 17, 24]. Our data seem to indicate that local progression of amyloid slows down after 6 years. This slowing-down of the disease may be caused by exhaustion of the underlying clonal plasma cells. A toxic effect on plasma cells of self-produced pre-amyloid might play a role [1]. In none of the series comprising ten or more patients with well-defined localized laryngeal amyloidosis and documented follow-up longer than 5 years, revision surgery was necessary after more than 7 years [8, 9, 12, 22, 23]. Thus a laryngological follow-up period of at least 7 years after the last surgical intervention is recommended in this disease.

Because localized laryngeal AL amyloidosis is regarded a benign disease, radical removal of all amyloid is not indicated. Suspension microlaryngoscopy [6, 7, 9] using cold instruments, CO_2_ laser and microdebrider are nowadays the surgical modalities of choice to treat laryngeal amyloidosis.

Our approach [3] is somewhere in between a “minimal excision technique” [9] and a rigorous debulking regimen [6]. It is guided by both symptoms (dysphonia, dysphagia, dyspnea) and signs (asymptomatic progression), as shown in Table 2. Excision is preferred. Debulking is performed instead of excision if voice, swallowing or breathing might be affected by radical resection. A need for possible revision is assessed during regular yearly follow-up (after 3 and 6 months in the first postoperative year) or when symptoms appear. Staged surgery per side is performed to prevent anterior webbing. For small deposits or deposits in the glottis, cold steel excision is favored. Excision with CO_2_ laser is preferred for bulky amyloid in the subglottis and supraglottis.

In older studies other surgical modalities have been described such as an external approach to the larynx [10] and total laryngectomy to remove the bulk of amyloid [11, 12]. The size and localization of amyloid sometimes necessitated a tracheotomy [8, 19–21] or endoluminal stents [8] to secure the airway. Fatal complications due to cannula-related death [8, 24] or hemorrhage with asphyxia [8] have been reported. Even a severe, recurrent, transglottic, bilateral bulky amyloid can now be mastered using multiple resections with CO_2_ laser and microdebrider [17], thereby preventing total laryngectomy as salvage surgery, as has been described in the past [12].

The main reason for surgical treatment and revision surgery in the study group was dysphonia. This corresponds with observations made in literature [6, 8, 9, 22]. The voice was impaired in more than 82 % initially, although in only 44 % the amyloid was located in the glottis. This discrepancy is explained by the observed mechanical obstruction of a supraglottic amyloid bulk at laryngostroboscopy. Such a bulk hampers formation of harmonics. In our study the voice is only mildly affected at the last visit. However, because of the observational nature of our study insufficient quantitative data were available to study a possible improvement of voice characteristics displayed in Table 3. Major limitations of this study were its observational nature and, although one of world’s largest series, the relatively small number of patients. Because of the long time span of our study, some preoperative voice characteristics were not available or not commonly known by referring physicians at the time of diagnostic endoscopy. This explains the lack of data on preoperative voice characteristics in most of the patients.

Amyloid can be present in a multifocal pattern in the larynx [23] or airways [22, 23]. It can even shift its location within the larynx, as we observed during our surgical interventions. We found multifocal and continuous deposits spanning more than one part of the larynx. Concomitant localized amyloid deposits outside the larynx, as in the eyelids (patient No. 12) or oropharynx (patient No. 2), have been reported in the nasopharynx [20], nasal sinus [15], tongue [23], oropharynx [10], tonsils [9, 20] and tracheobronchial tree [11, 12]. Two patients (No. 1 and No. 2) had a low grade plasma cell clone in the bone marrow. The multifocal pattern of laryngeal amyloid deposition, the concomitant multiple localizations outside the larynx, and the presence of a plasma cell clone in bone marrow all indicate that localized laryngeal amyloidosis is not really a strictly localized disease, but a local manifestation of a more widespread plasma cell dyscrasia.

## Conclusion

Treatment of choice in local laryngeal amyloidosis is calculated microlaryngeal surgery guided on symptoms and signs. After initial microlaryngeal surgery, amyloid recurred locally in all patients. Two-thirds of all patients needed revision surgery, half of them within 1 year after initial surgery. A second or third revision for amyloid was necessary in about 60 % of all patients within 6 years after the last surgery. Postoperative voice was only mildly affected in most patients. Follow-up of 7 years after last surgery is recommended because progression of the disease usually comes to a stop after 7 years.
